# Patient allocation method in major epidemics under the situation of hierarchical diagnosis and treatment

**DOI:** 10.1186/s12911-022-02074-3

**Published:** 2022-12-15

**Authors:** Yong Ye, Lizhen Huang, Jie Wang, Yen-Ching Chuang, Lingle Pan

**Affiliations:** 1 Institute of Public Health and Emergency Management, Taizhou University, Taizhou, 318000 Zhejiang China; 2 Business College, Taizhou University, Taizhou, 318000 Zhejiang China; 3 School of Electronics and Information Engineering, Taizhou University, Taizhou, 318000 Zhejiang China; 4 Zhejiang College of Security Technology, Wenzhou, 325000 Zhejiang China

**Keywords:** Hierarchical diagnosis and treatment, Patient allocation, Multi-objective planning, Major epidemics, The severity of patients’ conditions

## Abstract

**Objectives:**

Patients are classified according to the severity of their condition and graded according to the diagnosis and treatment capacity of medical institutions. This study aims to correctly assign patients to medical institutions for treatment and develop patient allocation and medical resource expansion schemes among hospitals in the medical network.

**Methods:**

Illness severity, hospital level, allocation matching benefit, distance traveled, and emergency medical resource fairness were considered. A multi-objective planning method was used to construct a patient allocation model during major epidemics. A simulation study was carried out in two scenarios to test the proposed method.

**Results:**

(1) The single-objective model obtains an unbalanced solution in contrast to the multi-objective model. The proposed model considers multi-objective problems and balances the degree of patient allocation matching, distance traveled, and fairness. (2) The non-hierarchical model has crowded resources, and the hierarchical model assigns patients to matched medical institutions. (3) In the “demand exceeds supply” situation, the patient allocation model identified additional resources needed by each hospital.

**Conclusion:**

Results verify the maneuverability and effectiveness of the proposed model. It can generate schemes for specific patient allocation and medical resource amplification and can serve as a quantitative decision-making tool in the context of major epidemics.

**Supplementary Information:**

The online version contains supplementary material available at 10.1186/s12911-022-02074-3.

## Introduction

Since the twenty-first century, new infectious diseases and other major epidemics, such as severe acute respiratory syndrome (SARS), Influenza A (H1N1), Ebola, and New Crown, have occurred frequently, seriously endangering the safety of human life and property. During large-scale epidemics, local healthcare systems will experience a large influx of patients, often with a scale that exceeds their capacity, leading to problems, such as crowding out of regional healthcare resources [1]. Saving infected patients is important to alleviate the epidemic.

Surge in medical demand and increased burden on hospital resources occur after a major epidemic. Ye et al. [2] pointed out that excessive medical resources are often required for treating unclassified patients, leading to additional medical and social problems. Gutierrez and Rubli [3] proposed that medical surge capacity can be improved by classifying patients and medical resources and triaging patients to different levels of medical institutions for treatment. Therefore, when the number of patients is far beyond the capacity of the medical system, the triage principle is needed to match the patients with effective medical resources. Hierarchical diagnosis and treatment refers to the transfer of patients with different conditions to corresponding medical institutions for treatment [4]. At present, relevant studies mainly focus on the allocation of routine outpatient surgery [5, 6], and no work has been conducted on the quantitative analysis and optimization of hierarchical diagnosis and treatment under major epidemics.

This study aims to develop an effective grading and triage model that depends on the condition of patients and the treatment capacity of medical institutions. The model will optimize the matching degree between patients and medical resources, reduce the depletion of medical resources, and determine feasible schemes for patient allocation and medical resource expansion in the medical network.

The contributions of this work can be summarized as follows:

- This paper provides healthcare managers with schemes for patient allocation and medical resource expansion to allocate limited hospital resources to infected patients and optimize their matching degree during a major epidemic.
- This paper will match patients with the right medical institution according to the concept of hierarchical diagnosis and treatment. The severity of the patient condition, the treatment capability of the medical institution, and the resources needed for different types of patients are considered.
- The hierarchical patient allocation model for diagnosis and treatment is universal and can be adjusted for different pandemics or medical structures.

The remaining parts of this paper are organized as follows. “Related work” section briefly reviews literature related to patient allocation, hierarchical diagnosis and treatment, healthcare allocation modeling, and simulation optimization under major pandemics. The hierarchical diagnostic patient allocation model, namely, NSGA-II, and the data from the COVID-19 epidemic in Shanghai, China, are presented in “Methods” section. “Results” section discusses the numerical results and overall performance of the case study and describes schemes that can help decision-makers determine patient allocation and expand medical resources. “Discussion” section provides the discussion. “Conclusion” section summarizes the results and limitations of the present work and recommends future improvement directions.

## Related work

Related research can be divided into three categories: patient allocation under major pandemics, hierarchical diagnosis and treatment, and healthcare allocation modeling and simulation optimization.

Studies related to major pandemics cover a wide range, including vaccine development [7–9], epidemic forecasting [10, 11], and mitigation strategy [12, 13]. The latter is an important research area and includes isolation [14], protection [15, 16], prevention and control [17], vaccination [18–20]. Rational allocation of patients is an important mitigation strategy when a large public health emergency occurs. A number of studies have been conducted on the allocation of patients under earthquake, hurricane, and other disasters [21–23]. Issues involving the allocation of patients and medical resources are also common, such as outpatient surgical allocation [24–26] and allocation of medical facilities [27]. However, a few studies have focused on patient allocation during major epidemics.

To our knowledge, only the following studies have discussed patient allocation protocols under pandemics. For example, Sun [28] addressed the patient allocation problem during an influenza pandemic by building an optimized model to minimize the distance of patients to hospitals. Tsai [29] applied a linear programming model for minimizing patients’ distance traveled to optimize patient allocation during a dengue epidemic. Both studies only considered the goal of distance traveled, although in reality, multiple goals should be optimized. Soroush [30] used a data envelope analysis approach to optimize the allocation of hospital beds during the COVID-19 pandemic. Patients in major epidemics often require multiple types of resources, but the study only considers the allocation of a single resource. Alternatively, none of these studies considered the degree of matching between the type of patients and the capacity of different medical institutions. During major epidemics, patients who do not receive matched treatment will have serious consequences [31].

Hierarchical diagnosis and treatment is a good tool used to assign patients to matched medical institutions for treatment [32]. In 1920, the concept of tertiary care was introduced in the UK [33]. The implementation of hierarchical diagnosis and treatment in the United States and other countries led to satisfactory results [34]. Relevant research focused on status analysis [35], institutional system challenge [36, 37], and diagnosis strategies for different diseases [38–40]. However, the quantification and optimization of hierarchical diagnosis and treatment under major epidemics has not been investigated yet.

Studies related to healthcare allocation modeling mainly differ in terms of optimization objectives and approaches. The objectives of optimization include distance [41], death number [42], risk [43], cost [31], and fairness [44]. The optimization methods include dynamic planning [45], random planning [46], and multi-objective planning [47, 48]. In reality, multiple conflicting optimization objectives exist, namely, multi-objective optimization. Aydin [49] considered three goals, namely, minimizing the total travel distance traveled, the maximum evacuation rate, and the risk to optimize ICU and non-ICU capabilities; this study also identified solutions by using a weighted sum method. Sun [28] dealt with the multi-objective problem by using a constraint method that moves all but one primary target to the constraint set. Zhang [50] used a multi-objective optimization approach with combination index weighted to obtain a general scheme for hospital inpatient bed allocation. These studies combine different objectives into a single objective, leading to a single objective problem.

A real-world multi-objective problem is usually needed to optimize multiple objectives simultaneously. Therefore, the best treatment is to find the most trade-off solution among all objectives [51]. Non-dominant ranking genetic algorithm (NSGA-II) is widely used in multi-objective optimization problems. The algorithm will assign adaptation to each individual according to Pareto ranking and crowding degree and will cover the solution as widely as possible [52].

The number of studies targeting the allocation of patients during major epidemics is limited. To our knowledge, these issues have only been discussed by Sun, Tsai, Soroush, et al. In this regard, the present work proposes a multi-objective model to optimize patient allocation with the highest matching benefit, minimal distance traveled, and optimal fairness. The diagnosis and treatment capacity of different medical institutions in the network of urban hospitals and the severity of patients’ conditions are considered to match them to appropriate medical institutions for treatment. This research also considers the capacity limitations of multiple healthcare resources. In particular, the NSGA-II algorithm is used to obtain an allocation scheme that balances all objectives.

## Methods

### Mathematical modeling and design

After a major epidemic, a large number of confirmed patients in the area are waiting for hierarchical diagnosis and will need to be dispatched to designated hospitals for treatment, depending on the severity of their condition. We assume that under the occurrence of a major epidemic, a city has $N_{i}$ epidemic areas, where patients are divided into *p* categories according to their degree of illness, and $N_{j}$ designated hospitals exist around the epidemic area, each with *q* categories of different treatment capacities. In the event of a major epidemic, the designated hospital centers provide matching means of treatment for all categories of patients in each epidemic area. In this paper, a multi-objective patient allocation scheme was designed by comprehensively considering the severity of patients’ conditions and classifying medical institutions according to their treatment capabilities. The optimization objectives included obtaining the highest matching benefit, the shortest distance traveled, and the most optimal fairness. The problem of allocation at different phases of an epidemic is also considered to ensure that more patients are admitted by expanding the medical treatment capacity of each hospital. Table 1 presents the symbols involved in the proposed model.

**Table 1:** Symbolic description of the model (Patient allocation method in major epidemics, China, 2022)

Ensemble	
$I$	The point ensemble in the epidemic area with demand for treatment, $I = \left\{ {i\left| {i = 1, \cdots ,N_{i} } \right.} \right\}$, where $N_{i}$ is the total number of epidemic areas
$J$	The ensemble of designated hospitals of patients, $J = \left\{ {j\left| {j = 1, \cdots ,N_{j} } \right.} \right\}$_,_ where $N_{j}$ is the total number of designated hospitals
$P$	The ensemble of patient states, $P = \left\{ {p\left| {p = 1, \cdots ,N_{j} } \right.} \right\}$, where $N_{p}$ is the the total number of patient's disease categories
$Q$	The ensemble of designated hospital level, $Q = \left\{ {q\left| {q = 1, \cdots ,N_{q} } \right.} \right\}$, where $N_{q}$ is the the total number of designated hospital level
$K$	The ensemble of medical resources, $K = \left\{ {k\left| {k = 1, \cdots ,N_{k} } \right.} \right\}$, where $N_{k}$ is the the total number of medical resources categories

Parameters	
$C_{jqk}$	Capacity of class *k* medical resources for hospital *j* of grade *q*
$A_{ip}$	Number of patients with Category *p* in epidemic area *i*
$D_{ij}$	Distance from epidemic area *i* to epidemic area *j*
$a_{pk}$	The number of class *k* medical resources required per class *p* patient
*M*	Unit allocation matching benefit matrix $M = [m_{pq} ]_{{N_{p} \times N_{q} }}$, Where $m_{pq}$ represents the allocation matching benefit coefficient for patients with Category *p* seen in q-level hospitals
*N*	Infinite quantity

Variables	
$r_{ip}$	Number of patients with Category *p* admitted in epidemic area *i*
$A_{ip}^{{ \star }}$	Number of patients in category P of epidemic area i waiting to receive treatment

Decision variables	
$v_{ipjq}$	Number of patients with Category *p* traveling from epidemic area *i* to q-level hospital *j*
$u_{ipj}$	Number of patients with Category *p* traveling from epidemic area *i* to designated hospital *j*
$v_{ipjq}^{{ \star }}$	Number of patients with Category *p* in epidemic area *i* assigned to *q*-level hospital *j*, and assigned to virtual capacity
$u_{ipj}^{{ \star }}$	Number of patients with Category *p* in epidemic area *i* who are assigned to hospital* j* and assigned to the virtual capacity

#### Objective function 1: allocation matching benefit

The correct level of patients should be assigned to the correct level of hospitals for treatment to ensure that each patient can receive the matching treatment methods and maximize the effectiveness of treatment resources. Patients of different categories will receive different treatment outcomes when they enter different levels of hospital (e.g., if severe patients enter third-class A hospital with Intensive Care Unit (ICU) ward and various perfect treatment methods, then they will get better treatment effect; by contrast, entering a makeshift hospital for isolation will produce poor effect). As such, allocation benefit coefficient $m_{pq}$ is defined as a measure of how well patients are matched to the corresponding hospital. It represents the allocation revenue coefficient for patients with category *p* seen in level *q* hospital. The value of $m_{pq}$ is larger when patients receive higher effectiveness when they visit a matching hospital, and it is smaller when patients receive lower effectiveness when they visit a hospital worse than the matching one. The value of $m_{pq}$ needs to be determined on the basis of clinical experience. In addition, patients who receive life-saving treatment will receive positive allocation effectiveness, and patients who do not receive life-saving treatment will have no allocation effectiveness. Therefore, the expression for consolidated allocated effectiveness is as follows:

1 $$f_{1} = \max \sum\limits_{q \in Q} {\sum\limits_{j \in J} {\sum\limits_{p \in P} {\sum\limits_{i \in I} {m_{pq} v_{ipjq} } } } }$$

#### Objective function 2: distance traveled

When major epidemics occur, decision makers often require that more patients can be treated in the fastest possible time [53]. In general, the longer the distance of the patient to the hospital, the longer the required transportation time, and the more likely the efficiency of treatment is reduced. Therefore, any means to admit and treat patients in the vicinity will be adopted to shorten the transportation time and improve the efficiency of treatment. Accordingly, this paper sets the objective function of the minimum distance traveled, and its expression is shown below:

2 $$f_{2} = \min \sum\limits_{j \in J} {\sum\limits_{p \in P} {\sum\limits_{i \in I} {D_{ij} u_{ipj} } } }$$

#### Objective function 3: principle of fair allocation

When a major epidemic occurs, every epidemic area is eager to receive life-saving treatment. However, the proximity strategy often fails to meet the needs of all epidemic areas. This paper defines the principle of fair allocation to balance the allocation of patients in various affected areas. In Adams’ theory of fairness, each person’s sense of fairness lies in the difference in comparison with others or with their own earlier comparisons; the smaller the difference is, the fairer they feel [54]. Patients in every affected area want to be allocated to more matched treatment. Therefore, the difference in the allocation matching benefit in each epidemic area can be narrowed down to achieve fairness. In this regard, this paper defines the principle of fair allocation.

##### Definition 1

(principle of fair allocation) Let $S_{i}$ be the average allocation matching benefit of the epidemic area *i*, whose expression is shown in Eq. (3).

3 $$S_{i} = {{\sum\limits_{p \in P} {\sum\limits_{j \in J} {\sum\limits_{q \in Q} {m_{pq} v_{ipjq} } } } } \mathord{\left/ {\vphantom {{\sum\limits_{p \in P} {\sum\limits_{j \in J} {\sum\limits_{q \in Q} {m_{pq} v_{ipjq} } } } } {\sum\limits_{p \in P} {\sum\limits_{j \in J} {\sum\limits_{q \in Q} {v_{ipjq} } } } }}} \right. \kern-\nulldelimiterspace} {\sum\limits_{p \in P} {\sum\limits_{j \in J} {\sum\limits_{q \in Q} {v_{ipjq} } } } }},\quad \forall i \in I$$

On this basis, the maximum average allocation matching benefit ($S_{\max }$) is obtained, and its expression is shown in Eq. (4).

4 $$S_{\max } = \mathop {\max }\limits_{i} S_{i}$$

 The difference between the average allocation matching benefit and the maximum average allocation matching benefit is defined as the maximum average allocation matching benefit deviation, whose expression is shown in Eq. (5).

5 $$\overline{S}_{i} = \left| {S_{i} - S_{\max } } \right|,\quad \forall i \in I$$

 Fair triage can be achieved by narrowing the difference in the average allocation matching benefit among epidemic areas to minimize the sum of allocation matching benefit deviation in each area. The principle of fair allocation can be expressed as Eq. (6).

6 $$f_{3} = \min \sum\limits_{i \in I} {\overline{S}_{i} }$$

 During the development of major epidemics, the number of patients is gradually increasing, and the relationship between the supply of medical resources and the demand of patients will also change from a state of “supply exceeding demand” to a state of “demand exceeding supply.” The patient allocation scheme for the two states will be studied. Thus, two models are developed for the following article.

#### Model 1: patient allocation model considering supply exceeding demand

Multi-objective function:

7 $$\{ f_{1} ,f_{2} ,f_{3} \}$$

Constraint:

8 $$\sum\limits_{i \in I} {\sum\limits_{p \in P} {a_{pk} } } v_{ipjq} \le C_{jqk} ,\quad \forall j \in J,q \in Q,k \in K$$

9 $$r_{ip} = A_{ip} ,\quad \forall i \in I,p \in P$$

10 $$r_{ip} = \sum\limits_{j \in J} {u_{ipj} } ,\quad \forall i \in I,p \in P$$

11 $$u_{ipj} = \sum\limits_{q \in Q} {v_{ipjq} } ,\quad \forall i \in I,\forall p \in P,\forall q \in Q$$

12 $$v_{ipjq} \ge 0,\quad \forall i \in I,\forall p \in P,\forall q \in Q$$

Constraint Formula (8) indicates the limit of medical treatment capacity. Constraint Eq. (9) means that the number of admitted patients is equal to the number of confirmed patients. Constraint Formula (10) is used to calculate the number of patients with category *p* admitted in epidemic area *i*. Constraint Formula (11) is used to calculate the number of patients with category *p* in epidemic area $i$ in designated hospital *j*. Formula (12) is a non-negative constraint.

#### Model 2: patient allocation model considering demand exceeds supply

During major epidemics, when the number of patients increases to a certain extent, the resources provided by medical institutions may not meet the needs of patients. At this point, various resource capacity limits for constraints (8) in Model 1 may not be fully met. Therefore, a feasible allocation scheme will not be obtained using Model 1. To address this problem, this work sets up a virtual capacity to absorb patients who will not be allocated to ensure the continuous generation of the allocation scheme. Patients have access to virtual resources only when all hospitals have insufficient resources. Therefore, patients allocated with virtual resources can be seen as patients waiting to receive treatment. The specific model is as follows:

Multi-objective function:

13 $$\left\{ \max \sum\limits_{q \in Q} {\sum\limits_{j \in J} {\sum\limits_{p \in P} {\sum\limits_{i \in I} {{{m_{pq} (v_{ipjq} + v_{ipjq}^{ * } )} \mathord{\left/ {\vphantom {{m_{pq} (v_{ipjq} + v_{ipjq}^{ * } )} {v_{ipjq}^{ * } }}} \right. \kern-\nulldelimiterspace} {v_{ipjq}^{ * } }}} } } } ,\min \sum\limits_{j \in J} {\sum\limits_{p \in P} {\sum\limits_{i \in I} {(D_{ij} u_{ipj} + ND_{ij} u_{ipj}^{ * } } } } ),f_{3} \right\}$$

Constraint:

14 $$\sum\limits_{p \in P} {\sum\limits_{i \in I} {a_{pk} v_{ipjq} } } \le C_{jqk} ,\quad \forall i \in I,q \in Q,k \in K$$

15 $$r_{ip} = \sum\limits_{j \in J} {u_{ipj} } = \sum\limits_{j \in J} {\sum\limits_{q \in Q} {v_{ipjq} } } ,\quad \forall i \in I,p \in P$$

16 $$A_{ip}^{ * } = A_{ip} - r_{ip} ,\quad \forall i \in I,p \in P$$

17 $$A_{ip}^{ * } = \sum\limits_{j \in J} {u_{ipj}^{ * } } = \sum\limits_{j \in J} {\sum\limits_{q \in Q} {v_{ipjq}^{ * } } } ,\quad \forall i \in I,p \in P$$

18 $$\sum\limits_{i \in I} {\sum\limits_{p \in P} {a_{pk} } } v_{ipjq}^{ * } = C_{jqk}^{ * } ,\quad \forall j \in J,q \in Q,k \in K;$$

19 $$A_{ip}^{*} \ge 0,u_{ipj}^{ * } \ge 0,v_{ipjq}^{ * } \ge 0,v_{ipjq} \ge 0,\quad \forall i \in I,\forall p \in P,\forall q \in Q$$

Multiple objective function (13) is obtained by Formula (7). In the first objective function, when the value of $v_{ipjq}$ is fixed, $v_{ipjq}^{ * }$ larger ${{m_{pq} (v_{ipjq} + v_{ipjq}^{ * } )} \mathord{\left/ {\vphantom {{m_{pq} (v_{ipjq} + v_{ipjq}^{ * } )} {v_{ipjq}^{ * } }}} \right. \kern-\nulldelimiterspace} {v_{ipjq}^{ * } }}$ is smaller. The smaller the $v_{ipjq}^{ * }$ is, the greater the ${{m_{pq} (v_{ipjq} + v_{ipjq}^{ * } )} \mathord{\left/ {\vphantom {{m_{pq} (v_{ipjq} + v_{ipjq}^{ * } )} {v_{ipjq}^{ * } }}} \right. \kern-\nulldelimiterspace} {v_{ipjq}^{ * } }}$ will be. Hence, the allocation matching benefit is maximized only when more small patients ($v_{ipjq}^{ * }$) are allocated to virtual capacity. The aim is to refuse to allocate patients with virtual capacity ($v_{ipjq}^{ * }$) as much as possible until all hospitals have insufficient resources. The $ND_{ij} u_{ipj}^{ * }$ part of the second objective function is identical. *N* is a very large value that is designed to make the distance between the hospital and the area as large as possible and to refuse to allocate to the virtual capacity of patients as much as possible. Constrained Eq. (14) represents the limitation of the capacity of various medical resources. Equation (15) represents the relationship of various variables. Constraint Eq. (16) is used to calculate the number of patients waiting for treatment. Equation (17) is the relationship between patient waiting to receive treatment and patient assigned to virtual capacity. Equation (18) is used to determine virtual capacity. Equation (19) is a non-negative constraint.

The model can determine whether hospitals need to increase medical resources in the situation of “supply exceeding demand” based on virtual capacity.

### NSGA-II algorithm

**Fig. 1:**
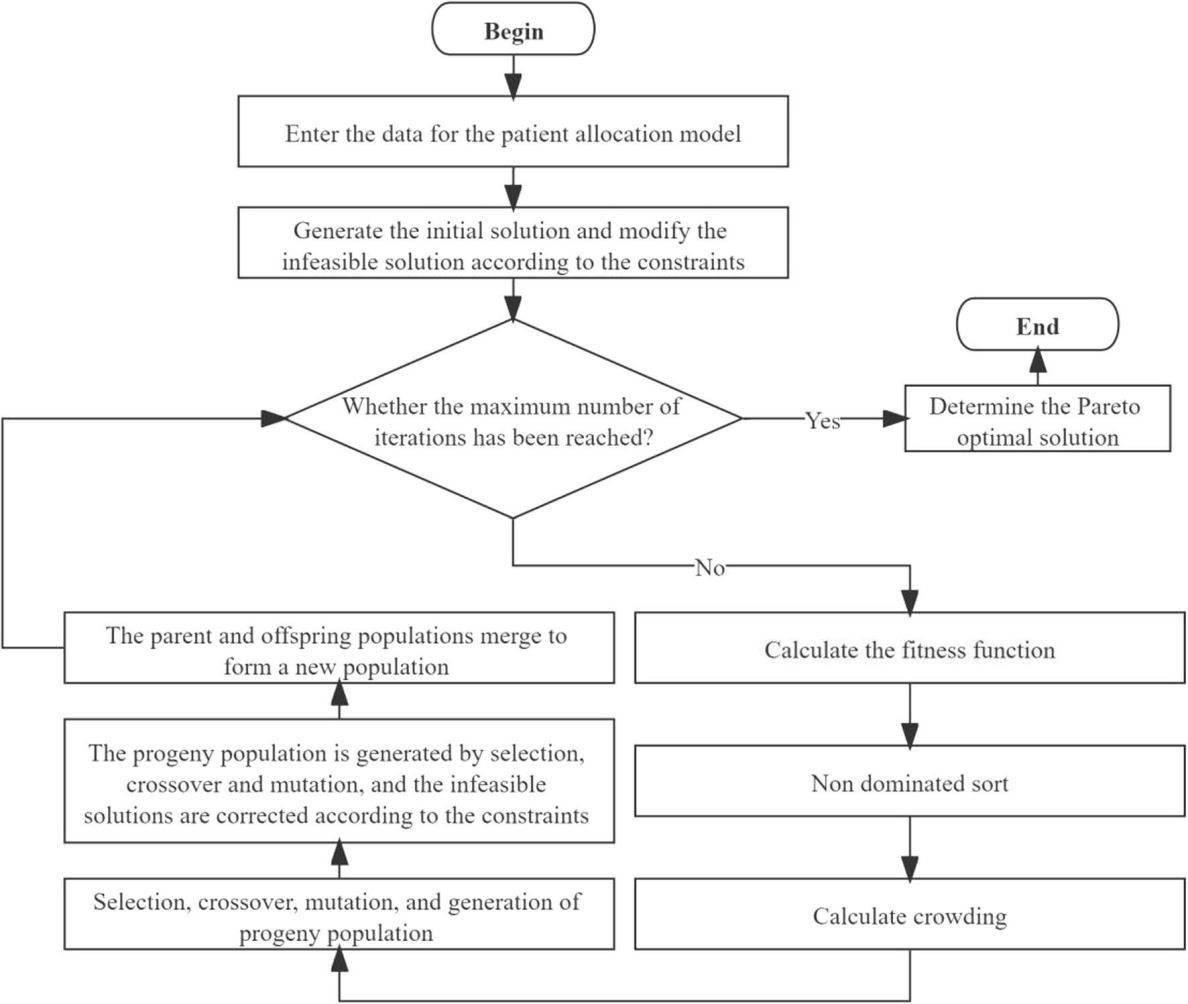
Flow chart of NSGA-II algorithm

Considering that the patient allocation model established is multi-constrained and multi-objective, it belongs to the NP problem, which cannot be solved by traditional algorithms. Genetic algorithm (GA) has strong global search ability, which can solve such problems well [51]. NSGA-II algorithm is obtained using GA genetic algorithm by combining non-dominant ranking and elite strategy [55]. It is a very popular and mature algorithm for solving multiple objectives [56]. Therefore, this work applies the NSGA-II algorithm to solve the model, and its flow chart is shown in Fig. 1.

(1) Initial population

The algorithm adopts positive integer coding, and each chromosome represents a feasible solution. If *N*_*p*_ patients in *N*_*i*_ epidemic areas need to be assigned to *N*_*j*_ hospitals, an initial population *P*_0_ with the scale of $N_{i} \times N_{p} \times N_{j}$ needs to be randomly generated. At the same time, the initial population is modified by the constraint of resource capacity.

(2) Fitness function

Because the objective function constructed in this paper is to maximize the distribution matching income (*f*_1_), minimize the journey distance (*f*_2_) and optimize the fairness of allocation (*f*_3_). The change trend of the three objective functions is different, which is not conducive to displaying Pareto curve intuitively. Therefore, the objective function is transformed into formulas (20), (21) and (22), respectively.

20 $$F_{1} = - f_{1}$$

21 $$F_{2} = f_{2}$$

22 $$F_{3} = f_{3}$$

(3) Fast non-dominated sorting

The Pareto grade $i_{(rank)}$ of individual *i* is determined according to the number of individual *i* dominated by other solutions in the population and the set of other solutions dominated by individual *i*.

(4) Crowding degree

In order to ensure the diversity of population, the crowding degree *i*_*d*_ is introduced to ensure that the algorithm can converge to a uniformly distributed Pareto surface [57]. Under a certain Pareto level, the crowding degree of individual *i* is calculated in formula (23).

23 $$i_{d} = \sum\limits_{m = 1}^{3} {\frac{{F_{m} (i + 1) - F_{m} (i - 1)}}{{F_{m}^{\max } - F_{m}^{\min } }}}$$

where $F_{m} (i + 1)$ represents the value of the objective function *m* of individual *i* + 1 before individual *i*; $F_{m} (i - 1)$ represents the value of the objective function *m* of individual *i* − 1 after individual *i*; $F_{m}^{\max }$ represents the maximum value of the objective function *m* under the Pareto level; $F_{m}^{\min }$ represents the minimum value of the objective function *m* under this Pareto level.

(5) Elite strategy

The parent population *P*_*t*_ and the offspring population *Q*_*t*_ produced by the parent population *P*_*t*_ are combined to compete together to produce the next generation $P_{t + 1}$.

(6) Genetic manipulation

Genetic operations include selection, crossover and variation.

- The selection operation is performed by comparing the Pareto grade $i_{(rank)}$ and the crowding degree *i*_*d*_ between individuals. If the Pareto grades of two individuals are different, take the individual with smaller grades; If two individuals are at the same level, take the individual with large crowding degree.
- The crossover operation uses a single-point crossover method [58].
- Single point random mutation is used for mutation operation [59].

### Application of simulation cases

This paper takes the most realistic possible data from Shanghai, China during the COVID-19 pandemic period to show how a patient allocation model based on hierarchical diagnosis and treatment helps decision makers to plan patient allocation during a major pandemic and validate the overall performance of the model. Although fully accurate data are not available, we believe that hospitals and managers will have more accurate information during the pandemic.

**Fig. 2:**
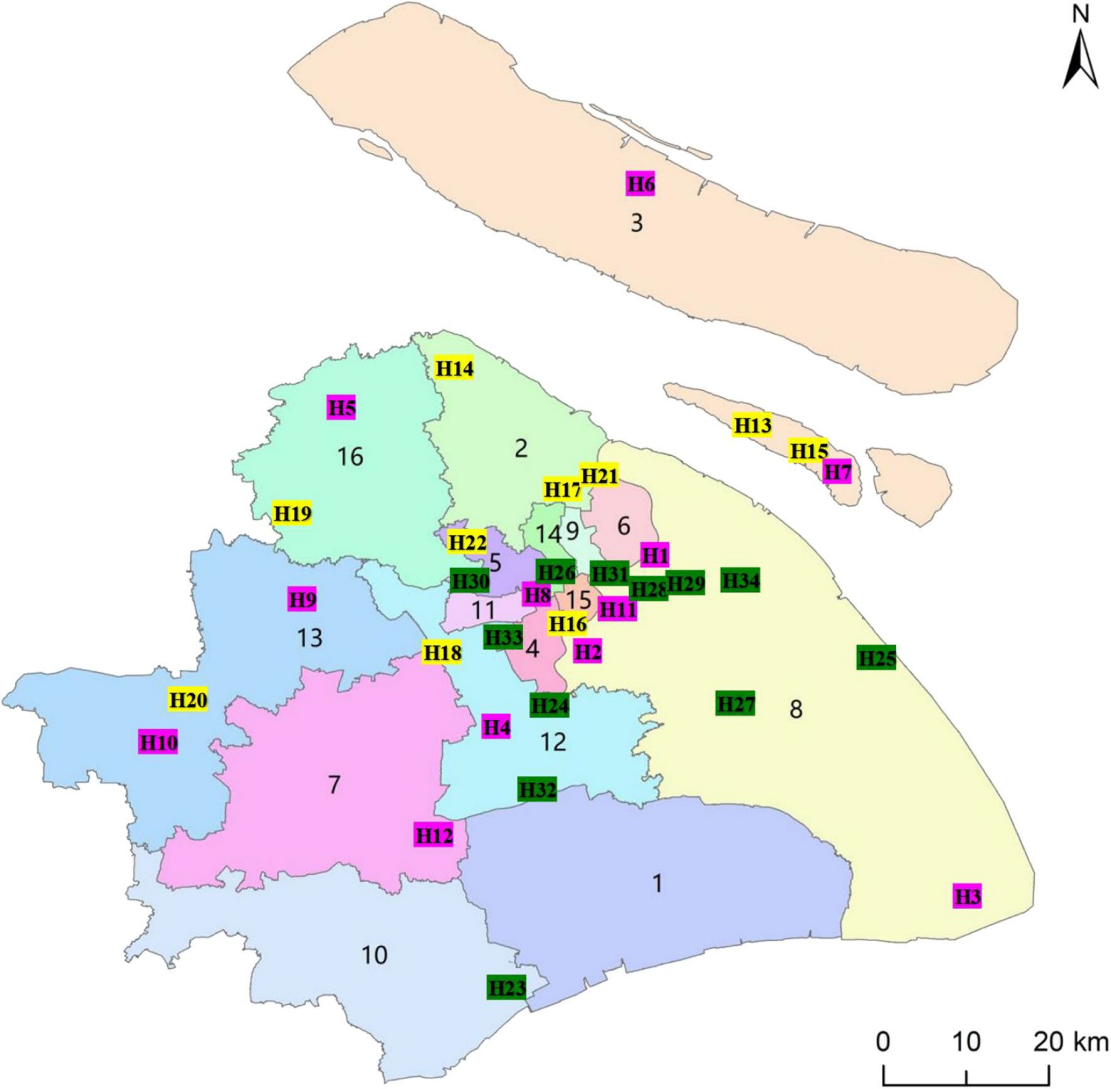
Map of Shanghai, China

The COVID-19 pandemic occurred in Shanghai, China from March 1 to May 24, 2022. As of May 24, the total number of infected people reached 648,334, and the highest number in one day was 23,370. For this case study, Shanghai was divided into 16 regions. Thirty-four hospitals and institutions (with incomplete data, because information is not available from some hospitals) were collected throughout Shanghai to treat infected patients. These hospitals and institutions include 12 3a-grade hospitals, 10 2a-grade hospitals, and 12 makeshift hospitals. Area numbers along with hospital location and level are shown in Fig. 2.

The distance of each area from each hospital (in km) is shown in Table 2. The data were collected from Google Maps.

**Table 2:** Distance of each area from each hospital

	A1	A2	A3	A4	A5	A6	A7	A8	A9	A10	A11	A12	A13	A14	A15	A16
Makeshift hospitals																
H1	48	27	89	16	26	8	44	4	10	74	19	24	51	17	12	44
H2	42	29	98	9	19	13	39	9	12	69	15	17	44	13	11	40
H3	47	80	125	71	82	65	88	60	67	70	76	70	102	73	71	102
H4	28	38	117	13	20	27	24	29	26	55	17	4	33	17	20	33
H5	62	32	118	40	26	37	54	43	34	83	31	40	42	32	36	3
H6	136	91	20	104	101	87	134	88	88	165	100	115	133	97	98	117
H7	80	36	44	49	45	32	79	32	32	109	44	59	78	41	42	61
H8	54	15	91	15	10	10	47	17	8	80	12	27	48	9	10	24
H9	40	37	116	21	18	29	30	30	29	60	20	16	3	20	23	30
H10	52	53	133	33	34	41	20	42	40	63	32	28	29	32	35	34
H11	40	25	99	8	14	13	36	11	13	66	11	16	43	9	7	36
H12	30	56	131	27	38	42	17	43	42	39	32	21	37	33	36	48
2a-grade hospitals																
H13	79	38	60	48	44	31	78	31	31	108	43	58	76	40	41	60
H14	56	14	102	34	20	24	52	31	22	82	25	34	50	23	24	16
H15	79	35	60	48	44	31	78	36	31	108	43	58	76	40	41	60
H16	39	24	98	7	17	11	35	10	11	65	10	15	42	8	6	36
H17	57	11	91	22	14	12	51	18	9	83	16	31	51	13	14	27
H18	45	34	113	23	16	27	29	33	26	59	16	20	27	18	26	25
H19	63	41	129	42	27	45	47	44	41	75	32	41	28	33	37	17
H20	66	67	147	47	48	55	37	56	55	69	46	42	19	46	49	48
H21	56	10	83	25	20	9	53	15	9	84	19	33	52	16	17	35
H22	43	23	99	15	3	20	40	20	17	70	11	20	40	9	13	21
3a-grade hospitals																
H23	28	85	160	56	67	74	45	72	73	8	61	49	60	64	67	76
H24	29	39	117	9	16	24	25	24	24	55	16	5	34	15	18	34
H25	50	54	105	46	65	41	65	36	43	78	51	46	78	49	47	78
H26	39	22	100	6	12	11	35	12	10	65	9	15	41	4	5	34
H27	37	48	101	26	37	24	45	19	26	66	31	25	57	28	27	58
H28	49	38	87	17	19	15	45	2	8	75	15	25	48	13	9	41
H29	45	23	93	13	18	18	41	4	7	71	15	21	48	12	7	40
H30	41	27	103	11	4	17	37	21	16	67	6	17	34	8	14	26
H31	42	22	100	6	11	8	38	8	7	68	8	18	40	4	2	33
H32	25	43	112	13	24	28	25	24	27	54	21	5	37	22	22	37
H33	34	28	105	3	11	18	30	17	17	60	7	10	36	8	12	36
H34	50	23	23	19	17	14	47	4	8	76	14	27	47	12	8	40

A represents the area, and H represents the hospital

Various resource capacities of each hospital are shown in Table 3. The number of beds and ICU in the 34 hospitals was compiled from the Shanghai Municipal Health Commission and the hospitals' official websites (these data are only an estimate based on the collated information). At present, no specific data of medical staff in each hospital are available, which is based on the proportion of the total number of medical staff * beds in the city.

**Table 3:** Number of medical resources in each hospital

Hospital	Bed	ICU	Staff
Makeshift hospitals			
H1	30,000	0	5000
H2	12,000	0	2000
H3	27,200	0	4533
H4	1000	0	167
H5	20,000	0	3333
H6	5400	0	900
H7	6000	0	1000
H8	1680	0	280
H9	80,000	0	13,333
H10	1000	0	167
H11	1332	0	222
H12	2600	0	433
2a-grade hospitals			
H13	300	6	336
H14	720	8	768
H15	300	6	336
H16	1400	10	1460
H17	1800	10	1860
H18	200	4	224
H19	500	6	536
H20	400	6	436
H21	1372	6	1408
H22	1000	6	1036
3a-grade hospitals			
H23	6000	40	6240
H24	1200	15	1290
H25	2000	25	2150
H26	4884	30	5064
H27	2000	17	2102
H28	4500	18	4608
H29	1000	15	1090
H30	1400	12	1472
H31	2400	14	2484
H32	4000	30	4180
H33	4600	30	4780
H34	2000	20	2120

Patients with COVID-19 in Shanghai can be divided into three categories: Type I patients are asymptomatic and who are in the incubation period and may develop symptoms in the future. Type II patients have mild symptoms. Type III patients have severe symptoms, and they are seriously ill and often need to be admitted to the ICU ward for treatment. The number of patients in each category for the two time periods for each area is shown in Table 4. On April 1, the epidemic was rising in Shanghai, and the number of patients was relatively small. On May 1, the peak of the epidemic was reached in Shanghai, and a large number of patients were infected. The data in the two time periods are divided into the allocation of “Supply exceeds demand” and “Demand exceeds Supply” scenarios. The data were obtained from the daily COVID-19 information released by the Shanghai Municipal Health Commission. The Commission has only reported the number of asymptomatic patients and confirmed patients and has no data on severe and mild patients. By considering the data from news reports, the proportion of severe patients is 1.2%, and the cumulative number of severe patients is determined according to the cumulative rate of confirmed patients * (1.2%). In practical application, the number of patients cannot be predicted in advance. The number of patients can only be estimated according to the prediction results of the infectious disease model.

**Table 4:** The number of infected patients

Area	On April 1			On May 1		
	Cumulative type A patients	Cumulative type B patients	Cumulative type C patients	Cumulative type A patients	Cumulative type B patients	Cumulative type C patients
A1	3509	53	0	13,592	464	6
A2	366	7	0	1572	49	0
A3	911	6	0	2993	39	0
A4	446	62	2	1851	213	3
A5	4078	250	2	11,907	721	7
A6	401	49	0	1045	151	0
A7	1644	260	2	3408	408	2
A8	8899	1213	18	21,261	2593	40
A9	6016	247	3	21,951	1055	14
A10	19,828	3451	55	59,286	8894	141
A11	123	29	0	261	55	0
A12	16,624	2185	35	41,307	5079	82
A13	2956	233	2	6238	624	4
A14	14,419	2178	36	48,740	6842	110
A15	18,759	305	2	53,246	1548	19
A16	23,114	954	16	69,178	3228	50

## Results

The results were obtained from MATLAB software based on the models, algorithms, and data described above.

### Parameter design

#### Model parameter

- *M:* Unit allocation matching benefit matrix. There are three types of patients, including type A patients, type B patients, and type C patients. There are three levels of hospitals, including makeshift hospitals (I), 2a-grade hospitals (II), and 3a-grade hospitals (III). For practical reasons, we want the sicker patients to be matched to higher-level hospitals. The value of $m_{pq}$ is larger when patients receive higher effectiveness when they visit a matching hospital, and it is smaller when patients receive lower effectiveness when they visit a hospital worse than the matching one. Therefore, we assume that the matching benefit of type A patients assigned to hospitals at levels I, II, and III is all 1. Type B patients assigned to both level II and level III hospitals have matching benefit of 3, while those assigned to level I hospitals have matching benefit of 1. A type C patient would have a matching benefit of 1 to a level I hospital, 3 to a level II hospital, and 5 to a level III hospital. The matching benefit of type C patients allocated to hospital at level I is 1. The matching benefit of type C patients allocated to hospital at level II is 3. The matching benefit of type B patients allocated to hospital at level III is 5. So, the matching benefit matrix of unit allocation is
  $$M = \left[ {m_{pq} } \right] = \left[ {\begin{array}{*{20}c} 1 & 1 & 1 \\ 1 & 3 & 3 \\ 1 & 3 & 5 \\ \end{array} } \right].$$
   The $m_{pq}$ set in this paper is only used to demonstrate the operability of the model. The specific value of $m_{pq}$ can be determined based on expert experience or the Delphi method.
  In addition, it should be noted that the model established in this paper is a hierarchical model considering the type of patients and the level of hospitals. To verify the validity of the model, the following results will compare the hierarchical model with the non-hierarchical model. The difference between non-hierarchical model and hierarchical model lies in the introduction of allocation matching benefit coefficient $m_{pq}$. $m_{pq}$ is introduced into the hierarchical model and removed from the non-hierarchical model. Because, for the non-hierarchical model without considering the type of patients and the level of hospitals, the model will no longer distinguish different patients from different treatment methods, and any patient who obtains any treatment methods will be judged as the same allocation benefit 1. In other words, in a non-hierarchical model, $m_{pq} = 1,\forall p,q$.
- $a_{pk}$: The number of class k medical resources required per class p patient. Suppose there are three medical resources that need to be allocated, including general hospital Bed, ICU, and Staff. Among them, type A patients need 1 ordinary hospital bed, 0 ICU, 1/6 medical staff, then $a_{Ak} = (1,0,1/6),k = Bed,ICU,Staff$. Type B Patients need 1 general hospital bed, 0 ICU, 1 medical staff, then $a_{Bk} = (1,0,1),k = Bed,ICU,Staff$. Type C patients need 0 general beds, 1 ICU, 3 healthcare staffs, then $a_{3c} = (0,1,6),k = Bed,ICU,Staff$.

#### Algorithm parameters

- Initial population size is 500.
- Maximum iteration is 100.
- Cross-over probability is 0.9.
- Mutation probability is 0.1.

### Contrast single-objective models and multi-objective models

**Fig. 3:**
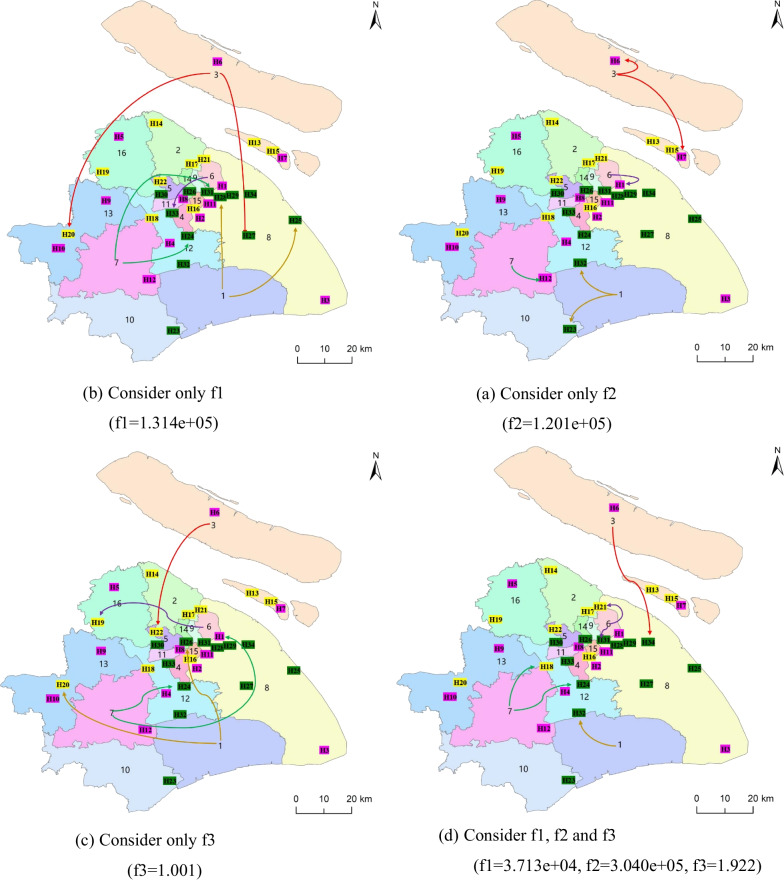
Patient allocation scheme for difference objective

To verify the feasibility of considering multiple objectives in the triage model, this work calculates the allocation schemes under different objective functions. For type II patients on April 1, the results are presented in Fig. 3, which shows the hospitals to which patients in each area should be assigned, depending on the objectives. For example, type II patients in area 1 were assigned to H25 (25th hospitals) and H28 (28th hospitals), considering only the objective function *f*_1_. In order to compare the allocation schemes under different objective functions more clearly, Fig. 3 only shows the allocation results of epidemic areas 1, 3, 6 and 7. See Additional file 1: Table S1 for detailed results. Considering that NSGA-II is an algorithm for solving multi-objectives, the single-objective problem cannot be implemented by the NSGA-II algorithm. The results for the single objectives in Fig. 3 are obtained by GA, and the multi-objective results are achieved by the NSGA-II algorithm. The NSGA-II algorithm is essentially derived from GA, and they only differ in selecting chromosomes to generate new populations. The two algorithms consider the same allocation pattern. Therefore, algorithmic differences do not affect the overall trends of different allocation schemes.

The results in Fig. 3 are compared between *f*_1_ (considering only the allocation matching benefit) and f2 (considering only the distance travelled) objectives. Patients under the *f*_1_ objective received more allocation matching benefit. Specifically, patients under the *f*_1_ objective were centrally assigned to high-level hospitals (e.g., 2a-grade hospitals and 3a-grade hospitals). Meanwhile, patients under the *f*_2_ objective were admitted to hospitals at all levels. However, patients under the *f*_1_ objective were assigned to further hospitals, such as A1 under the *f*_1_ objective to H25 (50.2 km) and H 28 (48.8 km), but patients under A1 and *f*_2_ objective were assigned to H23 (27.8 km) and H32 (25.4 km). The results under the *f*_3_ objective (considering allocation fairness only) were compared with those under *f*_1_ and *f*_2_ objectives. The *f*_3_ objective allocation scheme ensures that the average medical resources of patients are as small as possible, sacrificing the distance travelled (patients with A1 were assigned to H20 and H26 at distances of 65.9 and 38.7 km, respectively) and allocation matching benefit (more patients are assigned to the 2a-grade hospitals, allocation matching benefit low).

Therefore, models considering only a single-objective yielded imbalanced solutions. All the three objectives were considered to balance the degree of matching of patient allocation, the distance traveled, and the equity. Figure 3 shows that under multiple objectives (*f*_1_, *f*_2_, and *f*_3_), patients are more concentrated in 2a-grade hospitals and 3a-grade hospitals, while the patient distance traveled is relatively small (e.g., patients in A1 are assigned to H32 with a distance of 25.4 km). In addition, the objective value of the multi-objective model (*f*_1_ = 3.713e + 04, *f*_2_ = 3.040e + 05, *f*_3_ = 1.922) is relatively close to the objective value of each single-objective model. Therefore, considering the multi-objective model, the degree of allocation matching, the distance traveled, and fairness are relatively balanced.

### Contrast non-hierarchical and hierarchical models

The allocation scheme compares the graded model with the ungraded model to verify the triage model. The results of the assignment are shown in Tables 5 and 6. Table 5 shows the non-hierarchical and hierarchical allocation schemes in the “Demand exceeds supply.” Table 6 shows the non-hierarchical and hierarchical allocation scheme in the “Supply exceeds demand.” Tables 5 and 6 show the level of hospitals to which patients in each area were mainly assigned. For example, under the hierarchical model, type A patients in A1 were mainly assigned to grade I hospitals. Both tables have only provided a brief allocation protocol, and a detailed scheme will include the number of patients assigned to which level of which hospital.

**Table 5:** Non-hierarchical and hierarchical allocation scheme under “Demand exceeds supply” situation

	Type A patient		Type B patient		Type C patient	
	Hierarchical	Non-hierarchical	Hierarchical	Non-hierarchical	Hierarchical	Non-hierarchical
A1	I	I	II	I	III	III
A2	I	III	II	I	/	/
A3	I	I	II	III	/	/
A4	I	I	II	II	III	III
A5	I	II	III	I	III	II
A6	I	II	II	II	/	/
A7	I	I	II	I	III	III
A8	I	I	II	II	III	II
A9	I	II	II	I	III	III
A10	I	II	II	I	III	III
A11	II	I	II	III	/	/
A12	I	II	II	I	III	III
A13	II	I	II	II	III	III
A14	II	III	III	II	III	III
A15	I	II	II	III	III	II
A16	II	II	II	II	III	III

I represents Makeshift hospitals, II represents 2a-grade hospitals, and III represents 3a-grade hospitals

**Table 6:** Non-hierarchical and hierarchical allocation scheme under “supply exceeds demand” situation

	Type A patient		Type B patient		Type C patient	
	Hierarchical	Non-hierarchical	Hierarchical	Non-hierarchical	Hierarchical	Non-hierarchical
A1	II	III	II	III	/	/
A2	II	I	III	II	/	/
A3	II	I	II	III	/	/
A4	I	III	II	I	III	III
A5	II	I	II	II	III	III
A6	I	I	II	I	/	/
A7	II	III	II	II	III	III
A8	I	I	II	III	III	II
A9	II	I	II	I	III	II
A10	II	II	II	I	III	II
A11	III	III	III	II	/	/
A12	I	I	II	II	III	III
A13	II	II	II	I	III	III
A14	II	II	II	II	III	III
A15	II	II	II	I	III	II
A16	II	I	II	I	III	III

I represents Makeshift hospitals, II represents 2a-grade hospitals, and III represents 3a-grade hospitals

According to Table 5, when under the “supply exceeds the demand” situation, more type A patients in each region under the hierarchical model were assigned to grade I hospitals, more mild patients were assigned to grade II hospitals, and more severe patients were assigned to grade III hospitals. In reality, grade III hospitals are well equipped with high treatment levels. Grade II hospitals have general equipment and treatment level. Grade I hospitals are makeshift hospitals with few treatment means and low treatment level. In actual situations, the more serious the patient condition is, the higher the level of treatment will be. Thus, the results under the hierarchical model in Table 5 match the real-world considerations.

Under the non-hierarchical model, type A and B patients are assigned to grade III hospitals, while type C patients are assigned to grade II hospitals. Types A and B patients are crowding out the resources of severe patients.

The results in Table 6 are similar to those in Table 5, and the only difference is that more type A patients were assigned to grade II hospitals under the hierarchical model. The main reason is that the resources of hospitals are greater than the needs of patients in the “supply exceeds demand” situation. Thus, patients have the opportunity to choose a better hospital.

### Shortage of resources in “demand exceeds supply” situation

In the “Supply exceeds Demand” situation, medical resources can meet the needs of all patients. In the “demand exceeds supply” situation, medical resources cannot meet the needs of all patients. We need to consider how to expand medical resources to meet the needs of all patients. Model 2 is used to determine whether hospitals need to increase medical resources and how many resources are needed. In this case study, the additional resources required by each hospital in the “demand exceeds supply” situation are presented in Table 7. The results are used to guide how the hospitals in this case manage to increase the medical resources to ensure that all patients can be treated.

**Table 7:** Additional resources needed by the hospitals in the “demand exceeds supply” situation

Hospital	Bed	ICU	Staff	Hospital	Bed	ICU	Staff
H1	3010	0	576	H18	1137	0	139
H2	5349	0	603	H19	445	0	41
H3	748	0	136	H20	191	0	24
H4	631	0	82	H21	310	0	21
H5	1022	0	138	H22	1028	7	124
H6	2417	0	337	H23	447	0	41
H7	1344	0	184	H24	381	0	31
H8	4483	0	632	H25	457	0	42
H9	572	0	74	H26	1576	0	202
H10	2289	0	319	H27	549	0	55
H11	5710	0	808	H28	1610	20	364
H12	3309	0	465	H29	312	0	22
H13	346	0	41	H30	1198	10	148
H14	493	0	62	H31	432	0	39
H15	907	0	122	H32	712	20	94
H16	568	0	73	H33	1232	0	153
H17	737	0	97	H34	1934	33	253

## Discussion

Frequent pandemics of major epidemics seriously endanger the safety of human lives and property. Saving infected patients is an important means to alleviate the progression of the epidemic. However, after the pandemic of a major epidemic, the medical demand surges, and the burden of hospital resources increases, leading to run out of regional medical resources and other problems. In this regard, this work provides healthcare policy makers with useful decision-making tools for planning the allocation of patients and the expansion of medical resources under major epidemics.

Faced with the allocation of public health emergencies, decision-makers often consider the allocation of income [31], transportation benefit [41], and illness [40]. However, patients are more eager to have sufficient or more healthcare resources than others (i.e., fair allocation principle) [44]. Considering the perspective of decision makers and patients, the present work explores patient allocation strategies from three aspects: allocation matching benefit, distance traveled, and fair allocation principle. The objective function of the allocating matching benefit was used to facilitate patient access to more matched healthcare resources. The objective function of distance traveled was used to limit the proximity of patients to treatment. The objective function of the principle of fairness was used to ensure that the difference between the medical treatment received by patients in each epidemic area was as small as possible. When the model considering only a single objective obtained an uneven solution, the three objectives were considered simultaneously. The results indicate balanced degree of matching of patient allocation, distance traveled, and fairness.

**Fig. 4:**
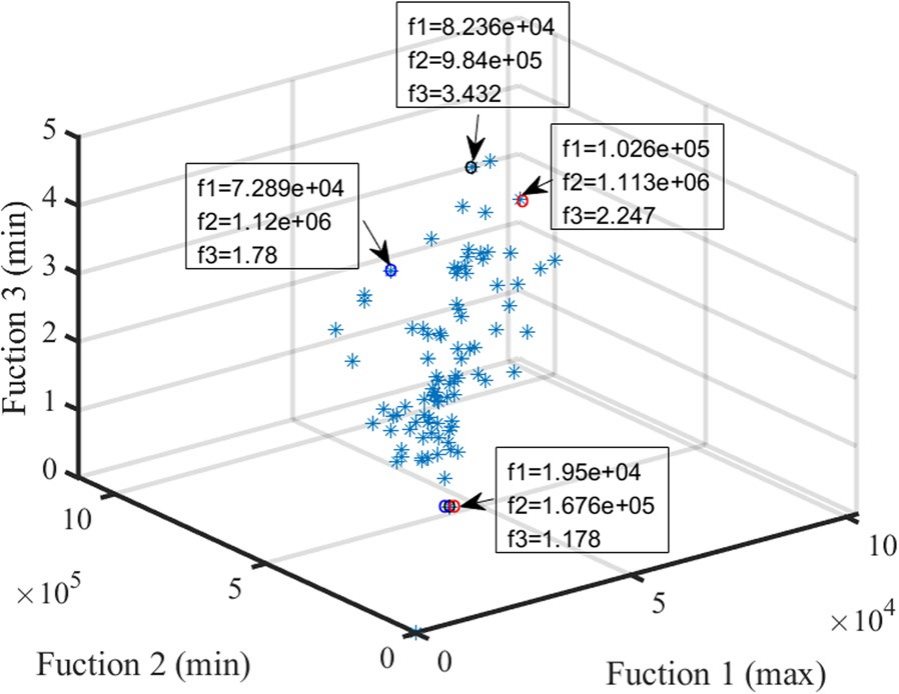
Pareto in “supply exceeds demand” situation

**Fig. 5:**
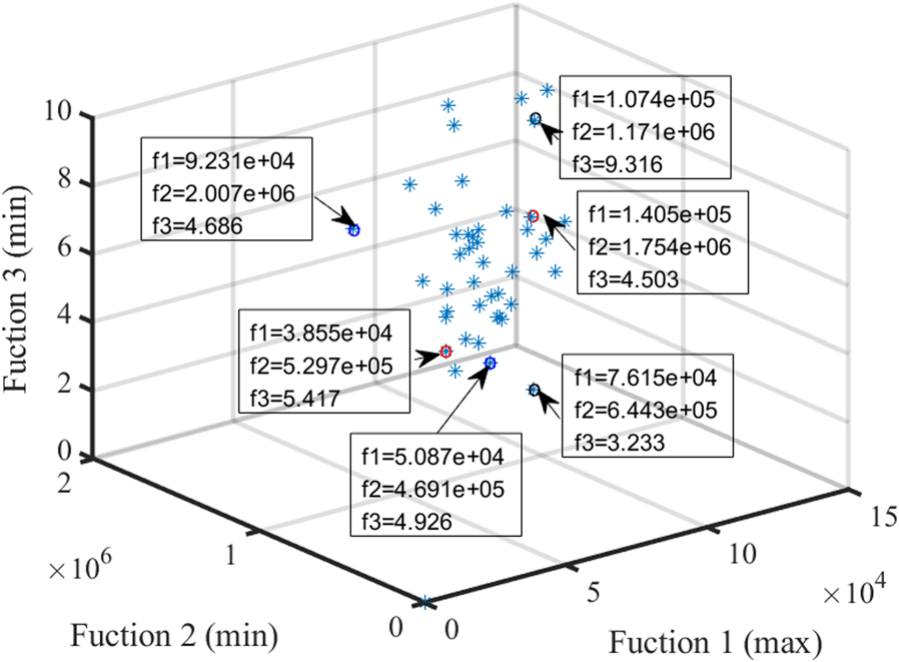
Pareto in “demand exceeds supply” situation

For multi-objective problems, no single solution that simultaneously reached the optimization of each objective can be found [51]. In this case, the objective functions were in conflict with one another. The optimization in obtaining a certain objective function was often at the expense of other objective functions. This work uses NSGA-II algorithm to generate different solutions for conflicting objectives. Figure 4 shows the Pareto solution in the “supply exceeds demand” scenario. Figure 5 shows the Pareto solution in the "demand exceeds supply" scenario. Although the number of solutions in Figs. 4 and 5 is different, it has an approximate change trend. Observing the overall change trend in Figs. 4 and 5, It can be seen that with the increase of allocation matching benefit, the distance traveled will increase as a whole, while the fairness of allocation will decrease. In other words, the increase of allocation matching benefits must be at the expense of increasing distance traveled or sacrificing the fairness of allocation. Therefore, users can find the best compromise according to their own preferences and the trade-off law of three objectives on Pareto surface. For example, when the requirement of allocation matching benefit is high, we should find the best solution from Pareto surface by increasing the distance traveled or reducing the fairness of allocation. Overall, the proposed NSGA-II algorithm provides multiple solutions and gives users greater flexibility to decide which solution best meets the requirements.

In addition, there may be endogeneity and highly correlated among the three objectives in the model. We can consider learning from Song et al.'s [60] practice to test the correlation between the objectives, and aggregate the objectives with significant positive correlations into a group to eliminate redundant objectives.

Hierarchical diagnosis and treatment is a good tool to assign patients to matched medical institutions for treatment. This work compares the allocation scheme of graded model. The results are as follows. (1) In the non-hierarchical model, resources are crowded, such as patients seizing the resources of patients with severe illness. (2) The scheme of the hierarchical model is the same as the concept of hierarchical diagnosis and treatment (matching to the corresponding treatment method according to the degree of the condition). The findings verified the effectiveness of the triage patient allocation model established in this paper.

As the epidemic develops, the number of patients in need of treatment will increase and will even exceed the maximum capacity of the medical system. Medical resources are the main reason to limit patients' treatment. Therefore, expanding the supply of medical resources should be considered to ensure that more patients can be treated. The model can be used to guide decision makers in deciding how to increase medical resources and ensure that all patients can be treated. In practice, the model cannot only discuss the supply scheme of the above medical resources but also add other medical resources (e.g., drugs) for discussion.

## Conclusion

After the occurrence of a major epidemic, the allocation of different categories of patients to the various hospitals is the core of the entire relief operation. This paper systematically considers the severity of patients' diseases, grade of hospital, allocation effectiveness, transportation distance and equity, and a patient allocation model in major epidemics is constructed with the objectives of the highest allocation effectiveness, the lowest transportation distance, and the equity of access to treatment for the patients, which is solved and analyzed by simulation data. The operability of the model is verified by results of the research.

The innovation of this paper is to consider the severity of patients’ conditions and the diversity of medical treatment capabilities, a patient allocation model in major epidemics is constructed by applying the multi-objective planning method. There are three objectives in this model, including the highest allocation effectiveness, the lowest transportation distance, and the equity of access to treatment for the patients in each epidemic area. This paper solves the problem of the allocation of various categories of patients under two scenarios: "supply exceeding demand" and "demand exceeding supply." Considering the fact that the hospital is at capacity and a large number of patients are not being admitted under "demand exceeding supply" scenario, admission and treatment demands of more patients can be satisfied by expanding medical treatment capacity. To maximize the utility of medical resources, the model provides decision schemes on how to expand the medical treatment capacity.

## Research limitations and future research directions

Many aspects in this paper can still be improved. Designing an applicable and efficient algorithm in the solving process will be the focus of the next research. In addition, the uncertainty of patient demands and the dynamics of the decision-making process will be the focus of the next research.

## Supplementary Information

**Additional file 1: Table S1.** Patient allocation scheme for different objective

## Data Availability

All data generated or analyzed during this study are included in this published article [and its supplementary information files].
