# Supplementary material for: Patient allocation method in major epidemics under the situation of hierarchical diagnosis and treatment
Source: BMC Med Inform Decis Mak. 2022 Dec 15;22:331. doi: 10.1186/s12911-022-02074-3 (PMC9753027; doi:10.1186/s12911-022-02074-3)
Supplement: Supplementary file 1 — Additional file 1: Table S1. Patient allocation scheme for different objective [file 12911_2022_2074_MOESM1_ESM.docx]

**TABLE S1** Patient allocation scheme for different objective

|  | f1  (f1=1.314e+05) | f2  (f2=1.201e+05) | f3  (f3=1.001) | f1、f2、f3  (f1=3.713e+04,  f2=3.040e+05,  f3=1.922) |
| --- | --- | --- | --- | --- |
| A1 | H25, H28 | H23, H32 | H20, H26 | H32 |
| A2 | H16, H19 | H21 | H19 | H17, H21 |
| A3 | H20, H27 | H6, H17 | H22 | H34 |
| A4 | H18, H25 | H16, H26 | H15 | H16, H33 |
| A5 | H18, H34 | H22 | H16, H21 | H22, H30 |
| A6 | H33 | H1 | H19 | H21, H31 |
| A7 | H24, H31 | H12 | H1, H24 | H18, H24 |
| A8 | H29 | H27, H34 | H11 | H16, H28 |
| A9 | H18, H23 | H28, H31 | H2, H14 | H21, H29 |
| A10 | H13, H19 | H23 | H5, H27 | H23 |
| A11 | H24, H33 | H30, H33 | H7, H28 | H30 |
| A12 | H27 | H4, H24 | H6, H13, H23 | H24 |
| A13 | H15 | H9 | H18 | H20 |
| A14 | H23,H29 | H26, H31 | H10, H33 | H16, H26 |
| A15 | H21 | H26, H31 | H13, H17 | H16, H22, H31 |
| A16 | H14, H32 | H5 | H2, H15, H30 | H14, H19 |
